# Association between Reflux Esophagitis Incidence and Palmar Hyperhidrosis

**DOI:** 10.3390/ijerph17124502

**Published:** 2020-06-23

**Authors:** Chun-Gu Cheng, Wu-Chien Chien, Chia-Peng Yu, Chi-Hsiang Chung, Chun-An Cheng

**Affiliations:** 1 Department of Emergency Medicine, Taoyuan Armed Forces General Hospital, National Defense Medical Center, Taoyuan 32549, Taiwan; doc50015@yahoo.com.tw; 2 Department of Emergency and Critical Medicine, Wan Fang Hospital, Taipei Medical University, Taipei 11696, Taiwan; 3 Department of Emergency Medicine, Xin Tai General Hospital, New Taipei 24262, Taiwan; 4 Graduate Institute of Life Sciences, National Defense Medical Center, Taipei 11490, Taiwan; chienwu@ndmctsgh.edu.tw; 5 Department of Medical Research, Tri-Service General Hospital, National Defense Medical Center, Taipei 11490, Taiwan; g694810042@gmail.com; 6 School of Public Health, National Defense Medical Center, Taipei 11490, Taiwan; yu6641@gmail.com; 7 Department of Neurology, Tri-Service General Hospital, National Defense Medical Center, Taipei 11490, Taiwan

**Keywords:** parasympathetic withdrawal, reflux esophagitis, palmar hyperhidrosis

## Abstract

The autonomic dysfunction in palmar hyperhidrosis (PH) includes not only sympathetic overactivity but also parasympathetic impairment. A decrease of parasympathetic tone has been noted in gastroesophageal reflux disease of neonates and adults. Patients with reflux esophagitis have a defective anti-reflux barrier. The association between reflux esophagitis and PH is deliberated in this article. The National Health Insurance Database in Taiwan was used. At first-time visits, PH patients were identified by the International Classification of Disease, 9th Revision, Clinical Modification disease code of 780.8 without endoscopic thoracic sympathectomy. Patients were matched by age and gender as control groups. The reflux esophagitis incidence was assessed using disease codes 530.11, 530.81, and 530.85. The factors related to reflux esophagitis were established by the Cox proportional regression model. The risk of reflux esophagitis in PH patients had a hazard ratio of 3.457 (95% confidence interval: 3.043–3.928) after adjustment of the other factors. We confirmed the association between reflux esophagitis and PH. Health care providers must be alerted to this relationship and other risk factors of reflux esophagitis to support suitable treatments to improve the quality of life of patients.

## 1. Introduction

Patients with palmar hyperhidrosis (PH) suffer from over-production of sweat in the palms induced by stress or exercise, which affects quality of life. A sympathetic overactivity of skin during stimulation by mental and thermal responses in PH patients has been shown [1]. The autonomic dysfunction in PH was found in a previous study to involve sympathetic overactivity combined with parasympathetic impairment [2]. The sympathetic overactivity in PH causes increased risk of cardiovascular disease and ischemic stroke compared with that of sympathectomy patients [3,4,5]. Given the parasympathetic disturbance present in both PH and reflux esophagitis, the relationship of two diseases was worthy of study.

Gastroesophageal reflux disease (GERD) is the backwash of gastric substances into the esophagus that causes symptoms with life-quality impairment [6,7]. The anti-reflux barrier is at the esophagogastric (EG) junction with the lower esophageal sphincter (LES) and curial diaphragm [8]. Past studies in neonates and adults showed that GERD events presented with decreased parasympathetic activity [9,10]. Disturbance of vagal activity could frequently relax the transient lower esophageal sphincter and reduce deglutition with delayed gastric empty [11]. The majority of GERD patients have no mucosal injury. Patients with reflux esophagitis, compared with non-erosive reflux, have anti-reflux mechanism impairment [12].

The relationship between reflux esophagitis and PH has not been discussed before. Both PH and reflux esophagitis include parasympathetic dysfunction; we designed a population-based retrospective cohort study to evaluate the possibility of an interaction between PH and sequent development of reflux esophagitis.

We assumed that increased sympathetic activity with parasympathetic impairment in PH patients may increase the incidence of long-term reflux esophagitis. We also checked other possible factors for health care providers, e.g., modifying lifestyle and adequate treatment, to reduce incidence.

## 2. Materials and Methods

### 2.1. Database

National health insurance in Taiwan started in 1995 and is run completely by the government. It includes approximately 99% of citizens in Taiwan. The longitudinal national health insurance research database (LNHIRD) contains one million randomly sampled patients and their records from 1999 onward. In LNHIRD, for personal privacy, data for each patient are encrypted with an original identification number. All outpatient and inpatient medical claims records in hospitals or clinics contain up to three outpatient disease codes and five inpatient disease codes according to the International Classification of Disease, Ninth Revision, Clinical Modification (ICD-9-CM). Our study assessed the occurrence of reflux esophagitis in PH patients by the LNHID from 1 January 2000 to 31 December 2013. Our study was approved by TSGHIRB-2-104-05-126.

### 2.2. Design

**Figure 1:**
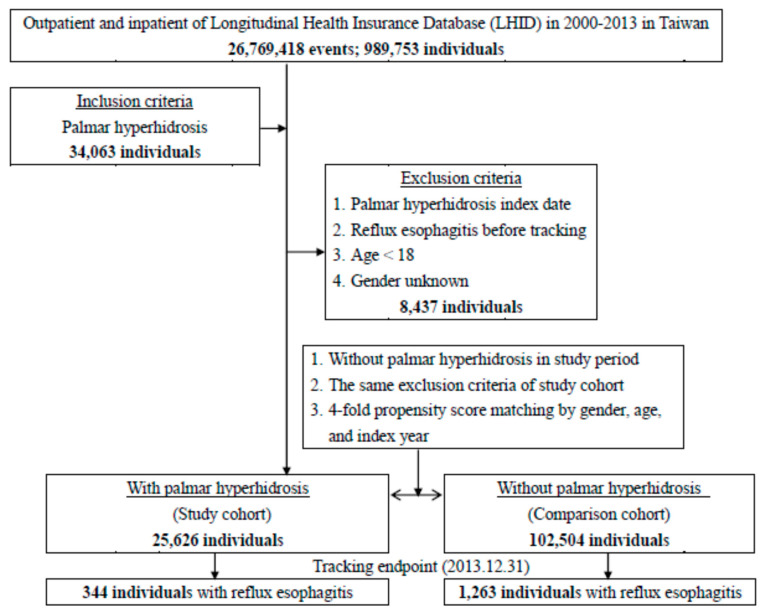
The flowchart of this study.

We found newly diagnosed PH cases (ICD-9-CM disease code 780.8) where endoscopic thoracic sympathectomies were not performed (operation code 05.29) from 1 January 2000 to 31 December 2013 in the LNHID of Taiwan. The disease code of PH were evaluated in previous studies [3,4]. The index date was assigned as the first visiting date for PH. Since reflux esophagitis includes an impairment of the anti-reflux barrier, compared with non-erosive esophagitis [12], we selected reflux esophagitis (ICD-9-CM disease codes 530.11, 530.81, and 530.85) as the outcome. The reflux esophagitis codes were assessed in a past study [13]. The second date was the diagnosis of reflux esophagitis in PH patients or the end time of study. We excluded (1) younger than 18 years old patients as they are often students in school preparing for entrance examinations with less time to seek out healthcare, (2) unidentified genders, and (3) PH and reflux esophagitis patients diagnosed before the study start date. The control group was designed by matching patients according to age, gender, and index date. The flow diagram of the study is presented in Figure 1.

The ICD-9-CM disease codes of comorbidities included hypertension (401–405), hyperlipidemia (272), obesity (278), diabetes mellitus (250), depression (296.2–296.3, 296.82, 330.4, 331), asthma (493), constipation (564.0), allergic rhinitis (477), obstructive sleep apnea (327.23, 780.51, 780.53, 780.57), osteoarthritis (715), osteoporosis (733), chronic obstructive pulmonary disease (491, 492, 494, 496), anxiety (300.1–300.3, 300.5–300.9), congestive heart failure (428), chronic kidney disease (580–589), and thyrotoxicosis (242).

### 2.3. Statistical Analysis

The categorical parameters were assessed by Chi-squared (X^2^) test and the continuous parameters by Student t-test for descriptive statistics according to the PH and non-PH groups, the statistical significance was set at a *p* value < 0.05. The relative factors of reflux esophagitis were assessed by Cox proportional regression with the hazard ratio (HR). The SPSS software version 21 (Asia Analytics Taiwan Ltd., Taipei, Taiwan) was employed for all analyses.

## 3. Results

**Figure 2:**
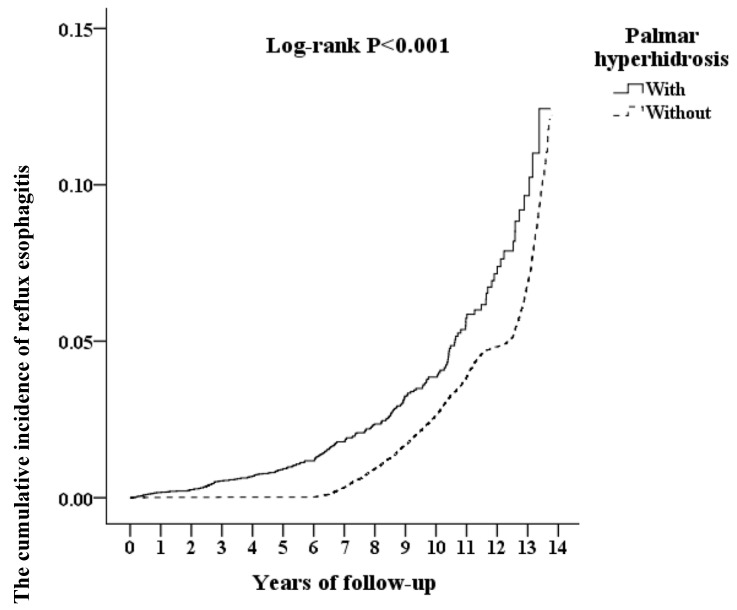
The cumulative incidence of reflux esophagitis by a Kaplan–Meier curve with log-rank test.

The occurrence of reflux esophagitis was tracked for a mean of 10.03 follow-up years. The incidence of reflux esophagitis was significantly higher in the PH group at 1.34% (344/25,626) than the non-PH group at 1.23% (1263/102,504) (log-rank *p* < 0.001, Figure 2).

The incidence of reflux esophagitis was higher in the PH group compared to the control group during spring and summer and in those with lower incomes, living in middle Taiwan or a higher urban area, and visiting a medical center or regional hospital. However, fewer comorbid conditions were noted in PH patients (Table 1).

The risk factors of reflux esophagitis were higher in PH patients with a HR of 3.457 (95% confidence interval (CI): 3.043–3.928), male gender with a HR of 2.376 (95% CI: 2.129–2.651), hypertension (HR: 1.73 (95% CI: 1.161–2.864)), diabetes mellitus (HR: 1.479 (95% CI: 1.271–1.714)), depression (HR: 1.512 (95% CI: 1.112–2.005)), chronic obstructive pulmonary disease (HR: 1.842 (95% CI: 1.34–2.537)), anxiety (HR: 2.031 (95% CI: 1.301–3.172)), and chronic kidney disease (HR: 1.363 (95% CI: 1.039–1.788)) after adjustment for the age of study group, income, level of health care, and season. (Table 2).

## 4. Discussion

To our knowledge, this is the first investigation about the sequent risk of reflux esophagitis in PH patients. The PH patients carried a higher risk of reflux esophagitis. The strength of our study is the population-based survey, which provided a large-sized sample of subjects and enabled us to assess the association between PH and reflux esophagitis. PH patients have excessive sweating in their palms due to a sympathovagal imbalance with high sympathetic activity and low parasympathetic function [2]. The potential mechanism of reflux esophagitis is through decreasing esophageal motility and delayed gastric emptying, increasing lower esophageal sphincter relaxation following reflux esophagitis incidents.

The prevalence of reflux esophagitis in hospital-based studies is 12–17.3% in Taiwan, and the majority of patients present with a mild degree and are free of reflux symptoms [14,15]. Certain epidemiologic factors, such as longevity, obesity, *Helibacter pylori*, and use of medications such as antihistamines, calcium channel blockers, anticholinergic agents, etc., are conducive to lower esophageal sphincter relaxation, and explain the increased prevalence of GERD [12]. However, other patients do not appear to have common factors, so discovery of another possible factor is important.

Peristalsis of the gastrointestinal tract is controlled by a parasympathetic nerve, and it enhances esophageal stomach motility with gastric emptying, and a sympathetic nerve inhibits esophageal contraction. The LES maintains tonic contractions of smooth muscles through neurogenic factors. The LES pressure is reduced by splanchnic sympathetic nerve stimulation and vagal-mediated tonic contractions [1]. A significant decrease of parasympathetic activity was noted during the prior reflux period in a neonate study [9]. More than 60% of GERD patients had transient lower esophageal sphincter relaxation (TLESR) [16]. One study found a higher frequency of TLESR and lower LES pressure in GERD patients compared with that of control in humans [17]. LES function is regulated by the enteric nervous system, with mechanoreceptors sensitization through the vagus nerve afferents from the stomach, signals then transfer into the nucleus tractus solitaries, whereas the caudal to the obex of dorsal motor nucleus of the vagus complex travel to vagal efferent fibers of the LES, resulting in relaxation.

GERD is multifactorial with a lack of balance between the aggressiveness of the refluxate into the esophagus and the failure of protective motility mechanisms or a defective valvular structure mechanism at the level of the EG junction [18,19]. It can impair vagal nerve responses by distal esophagus injury, particularly the afferent sensitization [20]. The anti-reflux barrier is localized at the EG junction and maintained by intrinsic LES pressure; the crural diaphragm is extrinsically compressed by the LES. Hiatal hernias reduce the barrier effect of the EG junction because the stomach is displaced proximally through the separation of the above two structures. The anti-reflux barrier is impaired in reflux esophagitis [12].

Comorbidities could assist health care providers in identifying better therapeutic approaches. Reflux esophagitis in PH patients occurred more frequently in spring–summer with possible over-sweating and sympathetic overactivity following higher air temperatures, higher urbanization areas, and higher care for their health conditions. Hypertension is the most common comorbidity in GERD [21], and our study discovered hypertension increased by 1.7-fold the risk of reflux esophagitis. The male sex showed as a risk factor for reflux esophagitis in a past study [22]. The risk factors for Chinese reflux esophagitis were hiatal hernia (odds ratio (OR):12.2), male sex (OR = 4.2), and chronic obstructive pulmonary disease (OR = 3.4) [9]. Chronic obstructive pulmonary disease caused negative pressure of the intrathoracic space, airway obstruction inducing TLESR, with anti-cholinergic medications reducing LES pressure [23]. Our study found male gender and chronic obstructive pulmonary disease carried 2.4- and 1.8-fold risks, respectively, of developing reflux esophagitis. One meta-analysis found greater risk (OR: 1.61) of GERD in diabetes mellitus patients compared with that of non-diabetes mellitus patients [24]. This study showed a similar result in that diabetes mellitus increased the risk of reflux esophagitis. Reflux esophagitis is also associated with anxiety, depression, and gastrointestinal symptoms [25]. The brain magnetic resonance images by esophageal stimulation presented higher activity of anterior insula and dorsal anterior cingulate gyri in negative mood patients than neutral patients [26]. Depression may induce reflux symptoms by decreasing the LES pressure, reducing the motility of the esophagus, increasing gastric acid secretions, or postponing the esophageal clearance of acid by the various stressors. Patients with major depression had an increased rate for GERD with an OR of 3.16 [13]. Our study found anxiety and depression increased by 2- and 1.5-fold, respectively, the risk of reflux esophagitis, which is lower than the previous study, possibly due to a different study design with PH and non-PH patients and a lower prevalence of around 40% of reflux esophagitis in GERD [12]. In Japan, 44.0% of patients with chronic kidney disease also have GERD [27]; our study found a 1.4-fold risk in chronic kidney disease with reflux esophagitis.

More GERD patients with comorbidities were elderly. Depression, asthma, and obesity were more common in middle-aged GERD patients. Allergic rhinitis was more common in young-aged GERD patients [21]. GERD incidence increased in in subjects over 40 years of age [28]. PH incidence decreased in older patients [29]. PH patients seek healthcare at a younger age, while a similar trend is not noticed with GERD. PH patients in our study had a mean age of 28 years with less osteoarthritis and osteoporosis. Previous studies observed that obesity with sympathetic overactivity and vagal withdrawal increased the risk of GERD; the autonomic impairment improved after weight reduction [30,31]. In contrast, our study did not find obesity increased the risk of reflux esophagitis, with a lower prevalence of obesity in youth of Asian ethnicity. Past studies showed that patients with asthma and sleep apnea had peristaltic dysfunction and increased risk of GERD [32,33], but our study did not find that asthma increased the risk of GERD, with lower prevalence in the young-aged group. Thoracoscopic sympathectomy decreases sympathetic activity and revises parasympathetic activity with a possible cardiac protection effect [34]. However, a past study found sympathectomy did not influence TLESR in animals [35]. We also surveyed PH after endoscopic thoracic sympathectomy and noted a similar result; no reduction in reflux esophagitis occurrence in humans. The potential reason may be the reduced cardiac sympathetic activity, such as a beta blocker effect, that did not affect TLESR of the EG junction without a reflux esophagitis preventive benefit with inadequate vagal stimulation after endoscopic thoracic sympathectomy. In addition, improved symptoms and parasympathetic activity in GERD were seen after proton pump inhibitor (PPI) treatment in a past study [36]. Future study is needed to evaluate the effect of PPI in PH patients. Reflux esophagitis can change to esophageal adenocarcinoma, but in our study only three patients with esophageal carcinoma (ICD9-CM: 150) were in the PH group and nine patients with esophageal carcinoma were in non-PH group, with statistical insignificant (*p* = 0.665).

There were some limitations in our study. First, because we detected reflux esophagitis by disease codes, the lack of endoscopy reports meant information on severity was unavailable. Further studies could collect endoscopy data to confirm the relationship between PH and different degrees of esophagitis. There was a lower prevalence of the two diseases in young adults according to seeking healthcare in the claim dataset; a registration study can improve this problem. Second, male sex, smoking habits, obesity, and hiatus hernia history carried risks of GERD development in a past study [10]. The information of patients such as body mass index, laboratory tests, and drinking and smoking history were unavailable in the dataset, which may lead to an overestimation of the reflux esophagitis risk of PH. Third, over-sweating caused young adults in Taiwan to seek healthcare help. Our study focused on Chinese ethnicity; the generalizability to other ethnic groups needs study in the future.

## 5. Conclusions

Our findings indicate that clinicians should understand the increased risk of reflux esophagitis for PH patients. GERD, which is a very common neurogastroenterologic problem, may be induced in PH patients due to autonomic dysregulation. In clinical practice, the patients with PH complained about annoying symptoms of GERD and considered consulting a gastroenterologist for treatment. In severer GERD cases with reflux esophagitis, the duration of PPI therapy may improve the underlying autonomic imbalance.

## Figures and Tables

**Table 1:** The characteristics of the palmar hyperhidrosis and control groups.

Variables	PH (%)	Non-PH (%)	*p*
**Total**	25,626	102,504	
Gender (Male)	12,131 (47.34)	48,524 (47.34)	0.999
**Age (years)**	28.16 ± 9.57	28.21 ± 9.79	0.999
Catastrophic illness	88 (0.34)	5245 (5.12)	<0.001 *
**Insured premium ($** **USD)**			<0.001 *
<600	19,900 (77.66)	76,864 (74.99)	
600–1167	3434 (13.4)	15,537 (15.16)	
≥1167	2292 (8.94)	10,103 (9.86)	
Hypertension	142 (0.55)	2456 (2.4)	<0.001 *
Hyperlipidemia	29 (0.11)	1661 (1.62)	<0.001 *
Obesity	4 (0.02)	51 (0.05)	0.017 *
Diabetes mellitus	75 (0.29)	2648 (2.58)	<0.001 *
Depression	38 (0.15)	309 (0.3)	<0.001 *
Asthma	32 (0.12)	682 (0.67)	<0.001 *
Constipation	10 (0.04)	132 (0.13)	<0.001 *
Allergic rhinitis	4 (0.02)	109 (0.11)	<0.001 *
Obstructive sleep apnea	2 (0.01)	45 (0.04)	0.005 *
Osteoarthritis	3 (0.01)	381 (0.37)	<0.001 *
Osteoporosis	0	84 (0.08)	<0.001 *
Chronic obstructive pulmonary disease	70 (0.27)	1200 (1.17)	<0.001 *
Anxiety	35 (0.14)	305 (0.3)	<0.001 *
Congestive heart failure	13 (0.05)	282 (0.28)	<0.001 *
Chronic kidney disease	15 (0.06)	1094 (1.07)	<0.001 *
Thyrotoxicosis	25 (0.1)	397 (0.39)	<0.001 *
**Season**			<0.001 *
Spring (3–5)	7819 (30.51)	26,354 (25.71)	
Summer (6–8)	8996 (35.1)	25,633 (25.01)	
Autumn (9–11)	4531 (17.68)	24,831 (24.22)	
Winter (12,1,2)	4280 (16.7)	25,686 (25.06)	
**Location**			<0.001 *
Northern	9915 (38.69)	42,264 (41.23)	
Middle	8714 (34)	28,514 (27.82)	
Southern	6081 (23.73)	25,825 (25.19)	
Eastern	834 (3.25)	5375 (5.24)	
Outlets islands	82 (0.32)	526 (0.51)	
**Urbanization level**			<0.001 *
1 (The highest)	8498 (33.16)	37,100 (36.19)	
2	11,186 (43.65)	41,349 (40.34)	
3	3783 (14.76)	9194 (8.97)	
4 (The lowest)	2159 (8.43)	14,861 (14.5)	
**Hospital levels**			<0.001 *
Medical center	6896 (26.91)	26,362 (25.72)	
Regional hospital	10,729 (41.87)	28,252 (27.56)	
Local hospital	8001 (31.22)	47,890 (46.72)	

PH: palmar hyperhidrosis. * *p* < 0.05.

**Table 2:** Risk factors of reflux esophagitis.

Variables	Crude HR	95% CI	*p*	Adjusted HR	95% CI	*p*
**Palmar hyperhidrosis**	2.903	2.569–3.279	<0.001 *	3.457	3.043–3.928	<0.001 *
**Gender (Female)**	Reference			Reference		
Male	2.167	1.454–2.667	<0.001 *	2.376	2.129–2.651	<0.001 *
**Age (years)**	1.002	0.948–1.068	0.245	0.997	0.992–1.001	0.161
Catastrophic illness	0.897	0.601–0.972	0.008 *	0.814	0.704–0.901	0.006 *
**Insured premium ($USD)**						
<600	Reference			Reference		
600–1167	1.024	0.467–1.464	0.802	0.959	0.672–1.368	0.816
≥1167	1.805	0.225–2.401	0.554	1.766	0.384–2.598	0.453
Hypertension	1.567	1.005–2.842	<0.001 *	1.730	1.161–2.864	<0.001 *
Hyperlipidemia	0.799	0.264–1.985	0.465	0.972	0.486–1.597	0.462
Obesity	2.297	0.446–5.012	0.246	2.208	0.985–4.95	0.154
Diabetes mellitus	1.454	1.121–1.77	0.001 *	1.479	1.272–1.714	<0.001 *
Depression	1.498	1.064–1.932	0.007 *	1.512	1.112–2.005	<0.001 *
Asthma	0.952	0.454–1.701	0.465	0.880	0.555–1.396	0.588
Constipation	0.882	0.498–1.985	0.703	0.934	0.67–1.588	0.879
Allergic rhinitis	0.468	0.105–2.345	0.154	0.537	0.119–2.888	0.101
Obstructive sleep apnea	9.565	0.099–78.452	0.875	11.245	0.167–197.752	0.786
Osteoarthritis	2.085	0.498–7.017	0.452	1.974	0.628–6.054	0.297
Chronic obstructive pulmonary disease	1.442	1.154–2.448	0.003 *	1.842	1.34–2.537	<0.001 *
Anxiety	2.989	1.498–3.902	<0.001 *	2.031	1.301–3.172	<0.001 *
Congestive heart failure	1.454	0.598–3.012	0.678	1.795	0.798–2.454	0.295
Chronic kidney disease	1.227	1.005–1.704	0.041 *	1.363	1.039–1.788	0.025 *
Thyrotoxicosis	0.996	0.124–1.642	0.564	1.088	0.157–1.516	0.215
**Season**						
Spring	Reference			Reference		
Summer	1.452	0.742–1.897	0.872	1.045	0.597–1.787	0.842
Autumn	0.804	0.512–0.911	0.002 *	0.779	0.678–0.893	0.003 *
Winter	0.667	0.334–0.876	0.004 *	0.564	0.487–0.642	0.001 *
**Urbanization level**						
1 (The highest)	0.795	0.642	0.053	0.889	0.743–1.063	0.197
2	0.701	0.611	0.025 *	0.861	0.754–0.984	0.028 *
3	0.685	0.572	0.001 *	0.676	0.575–0.796	<0.001 *
4 (The lowest)	Reference			Reference		
**Hospital levels**						
Hospital center	1.756	0.425–2.905	0.498	1.295	0.498–1.795	0.540
Regional hospital	1.015	0.375–1.785	0.811	0.884	0.331–1.454	0.735
Local hospital	Reference			Reference		

* *p* < 0.05. CI: confidence interval; HR: hazards ratio.
